# Impact of IL-27 on hepatocyte antiviral gene expression and function

**DOI:** 10.12688/wellcomeopenres.9917.1

**Published:** 2016-11-17

**Authors:** Narayan Ramamurthy, Sara Boninsegna, Rebecca Adams, Natasha Sahgal, Helen Lockstone, Dilair Baban, Emanuele Marchi, Paul Klenerman

**Affiliations:** 1 Peter Medawar Building for Pathogen Research, University of Oxford, Oxford, UK; 2 Sacrocuore Hospital, University of Padua, Padua, Italy; 3 Wellcome Trust Centre for Human Genetics, University of Oxford, Oxford, UK

**Keywords:** Hepatitis C virus, cytokines, inflammation, gene array, hepatocytes, hepatitis B virus, IL-27, CXCL10

## Abstract

*Background:* Interleukin (IL)-27 is a member of the IL-6/IL-12 family of cytokines. It is a potent cytokine, with potential antiviral impact, and has been shown to play a role in modulating functions of diverse cell types, including Th1, Th2, and NK and B cells, demonstrating both pro- and anti-inflammatory roles.  In hepatocytes, it is capable of inducing signal transducer and activator of transcription (STAT)1, STAT3 and interferon-stimulated genes.

*Methods: *To address its role in viral hepatitis, the antiviral activity of IL-27 against hepatitis C virus (HCV) and hepatitis B virus (HBV) was tested *in vitro* using cell-culture-derived infectious HCV (HCVcc) cell culture system and the HepaRG HBV cell culture model. To further investigate the impact of IL-27 on hepatocytes, Huh7.5 cells were treated with IL-27 to analyse the differentially expressed genes by microarray analysis. Furthermore, by quantitative PCR, we analyzed the up-regulation of chemokine *(CXCL)-10* in response to IL-27.

*Results:* In both HCV and HBV infection models, we observed only a modest direct antiviral effect. Microarray analysis showed that the up-regulated genes mostly belonged to antigen presentation and DNA replication pathways, and involved strong up-regulation of *CXCL-10*, a gene associated with liver inflammation. Overall, gene set enrichment analysis showed a striking correlation of these genes with those up-regulated in response to related cytokines in diverse cell populations.

*Conclusion: *Our data indicate that IL-27 can have a significant pro-inflammatory impact *in vitro*, although the direct antiviral effect is modest. It may have a potential impact on hepatocyte function, especially chemokine expression and antigen presentation.

## Introduction

Interleukin (IL)-27 is a member of the IL-6/IL-12 family of cytokines that includes IL-12 and IL-23. It is secreted by antigen presenting cells and has diverse impacts on host immune responses, including inducing the formation of antiviral Th1 cells ^1, 2^. It also inhibits Th2 humoral immune responses ^2^ and inhibits Th17 cells by blocking IL-17A production, and preventing up-regulation of RORγT ^3^. Recent data suggest it may also act on CD4+ T cells to negatively regulate function in tuberculosis ^4^.

IL-27 has also been described to possess antiviral functions. It inhibits HIV replication in CD4 cells and has been reported to control hepatitis C virus (HCV) in the cell-culture-derived infectious HCV (HCVcc) model, while in hepatitis B virus (HBV) infection, the virus activates IL-27 and interferon (IFN)λ1 and these co-ordinate to inhibit HBV replication ^5, 6^. In hepatoma cell lines, human and primary rat hepatocytes, the antiviral activity is thought to be induced by the induction of signal transducer and activator of transcription ( *STAT)1* and *STAT3*, leading to the induction of IFN regulated proteins, such as interferon response factor ( *IRF)-1, IRF-9*, guanylate binding protein 2 and myxovirus resistance A ^6, 7^.

In this study, we used the HCVcc model and the HepaRG cell line (an HBV infection model) to address the antiviral impact of IL-27 on hepatocytes, and used a genome wide microarray to analyse the interplay of different genes that were regulated after stimulation with IL-27.

## Methods

### HCV infection

Huh7.5 cells (Apath) were maintained in DMEM supplemented with Glutamax with 10% fetal calf serum (FCS), 100U/ml penicillin and 100μg/ml of streptomycin (cat# 15140122), and MEM non essential amino acids (cat# 11140035) at 37°C and 5% CO _2_. All reagents were GIBCO products from Thermo Fisher Scientific. IL-27 was obtained from R&D Systems Europe. Cells were stimulated with 100ng/ml IL-27 for 72 hrs at 37°C.

Huh7.5 cells were infected with genotype 2a chimeric HCV (MOI 0.05; J6/JFH1) (kindly provided by Apath) ^8^. Infected hepatocytes were treated with IL-27 at 100ng/ml and controls cultured with PBS as a control for up to 20 days. Cells were stimulated with IFNa (R&D systems Europe) at 1000u/ml as a positive control for antiviral activity. Immuno-fluorescence assays (IFA) were performed over this timecourse; cells were probed with 9E10 mouse monoclonal anti-non-structural protein 5 (NS5A) antibody (a gift from Dr. Charles Rice, Rockefeller University, USA) conjugated to Alexa 488 for the presence of HCV by fluorescence microscopy. Visualisation was performed using a Nikon Eclipse TE2000-U inverted microscope at x20 magnification using a Nikon DXM1200F camera. The software used for taking pictures was ACT-1 v2.63 and Adobe photoshop CS4 was used to count the infected cells.

### HBV infection

HepaRG cells (a gift from Dr. Nicole Zitzmann, University of Oxford) were cultured in Williams medium containing 10% FCS, 5μg/ml insulin and 50mM hydrocortisone at 37°C and 5% CO _2_. Cell differentiation was induced two weeks prior to infection by adding 2% v/v DMSO and epidermal growth factor (5ng/ml). Infectious HBV particles, for infection, were obtained from a culture of HepG2.2.15 cells (a gift from Dr. Nicole Zitzmann, University of Oxford). Briefly culture supernatants from HepG2.2.15 cells that were stably transfected with HBV plasmid were shaken in an orbital shaker overnight and following the addition of PEG8000 at a final concentration of 8%. The inoculum was pelleted by centrifugation at 13000xg for 30 mins, and reconstituted with sterile PBS.

To obtain viral load genome equivalents, HBV DNA was isolated from the inoculum using Invitrogen PureLink Viral DNA Mini kit (cat# 12280050), following the manufacturer’s instructions. The viral DNA was then quantified using forward: 5’-GGT CTC TTT CGG AGT GTG GA-3’; reverse: 5’-ATA GGG GCA TTT GGT GGT CT-3’ primers. qPCR was performed on a Roche 480 light cycler machine and using the Roche LC480 SYBR green master mix (cat# 04707516001) as below.

HepaRG cells were infected with the above inoculum with a genome equivalent of 20GE/cell overnight at 5% CO _2_ at 37°C. The infected HepaRG cells were treated with IL-27 (100ng/ml) or IFN-α (1000IU/ml) (R&D systems Europe).

## HBV ELISA

At day 7 post infection hepatitis B surface (HBs) antigen levels were assayed using MONOLISA HBs Ag Ultra Kit (Bio-Rad), according to the manufacturer’s instructions.

### RNA extraction and relative reverse transcription quantitative (q)PCR analysis

Total RNA was prepared using RNeasy kits (Qiagen). In-column DNAse treatment was performed. The quality of RNA was checked using Agilent Technologies 2100 Bioanalyser.

Two-step reverse transcription was performed using Superscript III Reverse Transcriptase (Invitrogen) and qPCR was performed using Roche Light Cycler 480 to detect CXCL10, low molecular mass peptide 7 ( *LMP7*) and transporter associated with antigen processing 1 ( *TAP1*). Primers were designed using the Roche Universal Probe Library system as follows *: CXCL10*, forward: 5’-GAA AGC AGT TAG CAA GGA AAG GT-3’ and reverse: 5’- GAC ATA TAC TCC ATG TAG GGA AGT GA-3’; *LMP7*, forward: 5’-CAA GTT CCA GCA TGG AGT GA-3' and reverse: 5’-TCA CCC GTA AGG CAC TAA TGT-3'; *TAP1*, forward: 5’-GCA AGA AAT AAA GAC ACT CAA CCA-3' and reverse: 5’-CCC ACT TTC AGC AGC ATA CC-3'; *GAPDH* forward: 5’-AGC CAC ATC GCT CAG ACA C-3’ and reverse: 5’-GCC CAA TAC GAC CAA ATC C-3’.

Relative gene expression was calculated using the comparative cycle threshold method, as described previously ^9^.

### Gene expression and statistical analysis

Gene expression profiles were obtained by hybridising the samples to GeneChip Human Gene 1 STU Arrays (HuGene-1_0-st-v1; Affymetrix), according to the manufacturer’s instructions. Statistical testing was performed using Linear Models for Microarray Analysis (LIMMA) package ( http://bioconductor.org/packages/release/bioc/html/limma.html) ^10^. Raw p-values were corrected using the false discovery rate controlling procedure of Benjamini and Hochberg ^11^. Following this correction, adjusted p-values <0.01 were considered significant. Gene annotation was added to the final probe list from the NetAffx™ Analysis Center ( https://www.affymetrix.com/analysis/index.affx).

We used the online Database for Annotation, Visualization and Integrated Discovery (DAVID v6.8) bioinformatics database ( https://david.ncifcrf.gov/) to analyze pathways ^12^. Gene set enrichment analysis (GSEA) from the Broad Institute ( http://software.broadinstitute.org/gsea/msigdb/) was used to assess significant enrichment of immunological signature gene sets in IL-27 up-regulated genes.

## Results

### Antiviral studies

**Figure 1.**
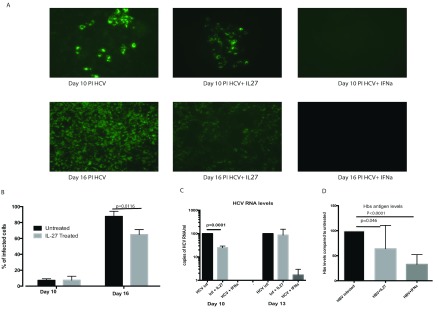
Impact of IL-27 in HCV replication *in vitro*. Huh7.5 cells were infected with HCV and treated with IL-27 (100ng/ml). ( **A**) Immuno-fluorescence assays were performed during the experimental period to assess for antiviral activity. ( **B**) Infected cells were counted and the percentage of infected cells compared to the control untreated well. ( **C**) Supernatants from cells infected with HCV and control untreated wells were analysed by real time RT-PCR and represented as copies of HCV/ml of supernatant. ( **D**) HBs antigen levels as assessed by ELISA. Results from three independent experiments are shown (p-value assessed using t-test). IL, interleukin; PI, post-infection; HCV, hepatitis C virus; IFN, interferon; HBs, hepatitis B surface antigen; HBV, hepatitis B virus; Inf, infection.

In the HCVcc model, it was previously demonstrated ^6^ that IL-27 had antiviral properties. Using the same model, we have demonstrated that there is no significant reduction in viral infectivity at 10 days post treatment with IL-27 at 100ng/ml, as observed by an immuno-fluorescence assay (IFA) of infected hepatocytes ( Figure 1A). However, at 16 days a modest impact of IL-27 on infection was observed with 73.8% of cells infected compared to controls (normalized to 100%; p=0.0116 t test; Figure1B). Analysis of viral RNA at day 10 revealed a small difference between treated and untreated cells ( Figure 1C); however, the effect is overall limited compared to the IFNγ positive control ( Figure 1B) and was not significantly different when measured at a later time point (day 13).

Similarly, at day 7 post-infection, HepaRG cells infected with HBV showed modest reductions in HBsAg levels after treatment with IL-27 ( Figure 1D).

### Gene expression studies

To further understand the impact of IL-27 on hepatocytes, we next addressed the gene expression changes occurring during IL-27 treatment. Microarray analysis showed that IL-27 significantly induced (>two-fold) a total of 446 genes, while 129 genes were down-regulated in Huh 7.5 cells, 72 hours post-stimulation ( Supplementary Table 1). Table 1 shows the top 20 differentially regulated genes obtained.

**Table 1.** Table showing top 20 genes differentially regulated in Huh7.5 cells in response to 100ng/ml of IL-27 at 72 hrs post stimulation.

Up-regulated genes			Down-regulated genes	
Gene symbol	Gene title		Gene symbol	Gene title
SLC6A14	solute carrier family 6 (amino acid transporter),		USP17L6P	ubiquitin specific peptidase 17- like 6
LOC100289612	arsenic transactivated protein 1		UIMC1	ubiquitin interaction motif containing 1
RABL2A	RAB, member of RAS oncogene family- like 2A		FOS	FBJ murine osteosarcoma viral oncogene homolog
SPCS2	signal peptidase complex subunit 2 homolog		EGR1	early growth response 1
UBD	ubiquitin D		JUN	jun oncogene
DKK1	dickkopf homolog 1 (Xenopus laevis)		RPPH1	ribonuclease P RNA component H1
CXCL10	chemokine (C-X-C motif) ligand 10		TMEM191A	transmembrane protein 191A
ASPM	asp (abnormal spindle) homolog, microcephaly associated (Drosophila)		PIK3IP1	phosphoinositide-3-kinase interacting protein 1
NTS	neurotensin		RNU22	RNA, U2 small nuclear 2
NME1	non-metastatic cells 1, protein (NM23A		GADD45B	growth arrest and DNA-damage- inducible, beta
MCM10	minichromosome maintenance complex component 10		SMA4	glucuronidase, beta pseudogene
ORM2	orosomucoid 2		FMO1	flavin containing monooxygenase 1
ESCO2	establishment of cohesion 1 homolog 2 (S.cerevisiae)		LOC642838	similar to hCG1742442
CDC6	cell division cycle 6 homolog (S. cerevisiae)		BTG2	BTG family, member 2
CDC45L	CDC45 cell division cycle 45-like (S. cerevisiae)		GDF15	growth differentiation factor 15
LYZ	lysozyme (renal amyloidosis)		RFC1	replication factor C (activator 1) 1, 145kDa
CDC25A	cell division cycle 25 homolog A (S. pombe)		PLK2	polo-like kinase 2 (Drosophila)
MCM4	minichromosome maintenance complex component 4		FOSB	FBJ murine osteosarcoma viral oncogene homolog B
LCORL	ligand dependent nuclear receptor corepressor-like		DKFZP564O0823	prostatic androgen-repressed message-1
DTL	denticleless homolog (Drosophila)		LOC100128868	testin-related protein TRG
GINS1	GINS complex subunit 1 (Psf1 homolog)		JUNB	jun B proto-oncogene
HIST1H2AB	histone cluster 1, H2ab		DIO1	deiodinase, iodothyronine, type I
DHFR	dihydrofolate reductase		DUSP1	dual specificity phosphatase 1

Amongst the top 20 hits in our data were a number of signaling pathway genes notably Rab-like protein 2A ( *RABL2a*) (a GTPase that mediates signal transduction), neurotensin ( *NTS*) and signal peptidase complex subunit 2 homolog ( *SPCS2*). Cell cycle proteins, e.g. the calmodulin binding *ASPM* gene, *CDC6*, which regulates DNA replication, along with DNA replication checkpoint gene, *CDC45L*, were also up-regulated. Other relevant up-regulated genes were those involved in inflammation, including *CXCL-10*, which is known to be IFN responsive, orosomucoid ( *ORM)-1/2* and lysozyme ( *LYZ*) ( Table 1).

The DAVID program was used to analyse the data set to identify pathways that are differentially regulated in response to stimulation by IL-27. Kegg functional annotation analysis within this program ( Supplementary Table 2A) of the up-regulated genes, showed high fold-enrichment and significance in genes involved in DNA replication, the cell cycle and homologous recombination. Gene functional classification classified genes into 14 clusters, the largest, with a high enrichment score, included genes involved in the cell cycle, cell division, DNA replication, the spliceosome, and nucleic acid metabolism, and a strong signal from the proteasomal signaling pathway ( Supplementary Table 2B).

Analysing the down-regulated genes, gene functional classification showed only one gene cluster with a significant score, which included transcriptional regulators v-maf musculoaponeurotic fibrosarcoma oncogene homolog *MAF*, early growth response 1 ( *EGR1*) and Jun- D proto-oncogene ( *JUND*). *NR1D1*, a nuclear receptor and transcription repressor involved in circadian regulation, which is normally highly expressed in the liver, was also reduced in response to IL-27. Target genes of *NR1D1* are ApoA1 and ApoCIII and anti-fibrinolytic factor PAI-1, while *NR1D2* is involved in the control of lipid and energy homeostasis in skeletal muscle ^13^.

### Gene set enrichment analysis

**Figure 2.**
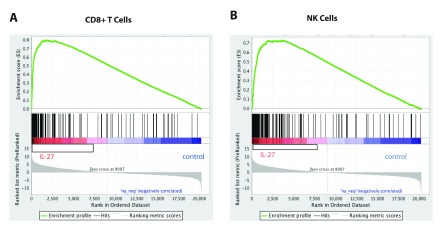
Enrichment of immune signatures in IL-27 treated hepatocytes. ( **A**) Representative analysis of our genes when compared by gene enrichment analysis against the immunological signals from the Broad Institute gene enrichment database showed genes up-regulated with IL-27 were also those that were found up-regulated in CD8+ T cells (GSE 15750; https://www.ncbi.nlm.nih.gov/geo/query/acc.cgi?targ=self&form=html&view=brief&acc=GSE15750) and ( **B**) NK cells in response to IL-15 (GSE 22886; https://www.ncbi.nlm.nih.gov/geo/query/acc.cgi?targ=self&form=html&view=brief&acc=GSE25616). The steep upslope of the curve (leading edge) indicates strong, statistically robust enrichment of relevant genes. IL, interleukin.

Gene set enrichment analysis (GSEA) was used to understand the significance of IL-27 induced gene expression, in comparison with other published experimental datasets. Genes differentially regulated in response to IL-27 were compared against a list of immunological signatures from the Broad Institute GSEA database. We found linked gene expression sets in experiments using related cytokines on distinct cell types. Figure 2 shows representative enrichment plots of relevant gene signatures from datasets with significant correlation to our experiments on Huh7.5 cells (CD8+ T cells and NK cells following treatment with IL-15, a related cytokine *in vitro)*. Supplementary Table 3 give a more comprehensive list (top 20 most significant enriched gene sets) of experiments that have similar differentially up (S-2A) or down (S-2B) regulated genes, respectively. Overall, this shows clear overlapping gene sets between genes induced in our experiments and cellular activation by lymphocytes in response to related cytokines.

### Quantitation of CXCL-10, TAP-1 and LMP7

**Figure 3.**
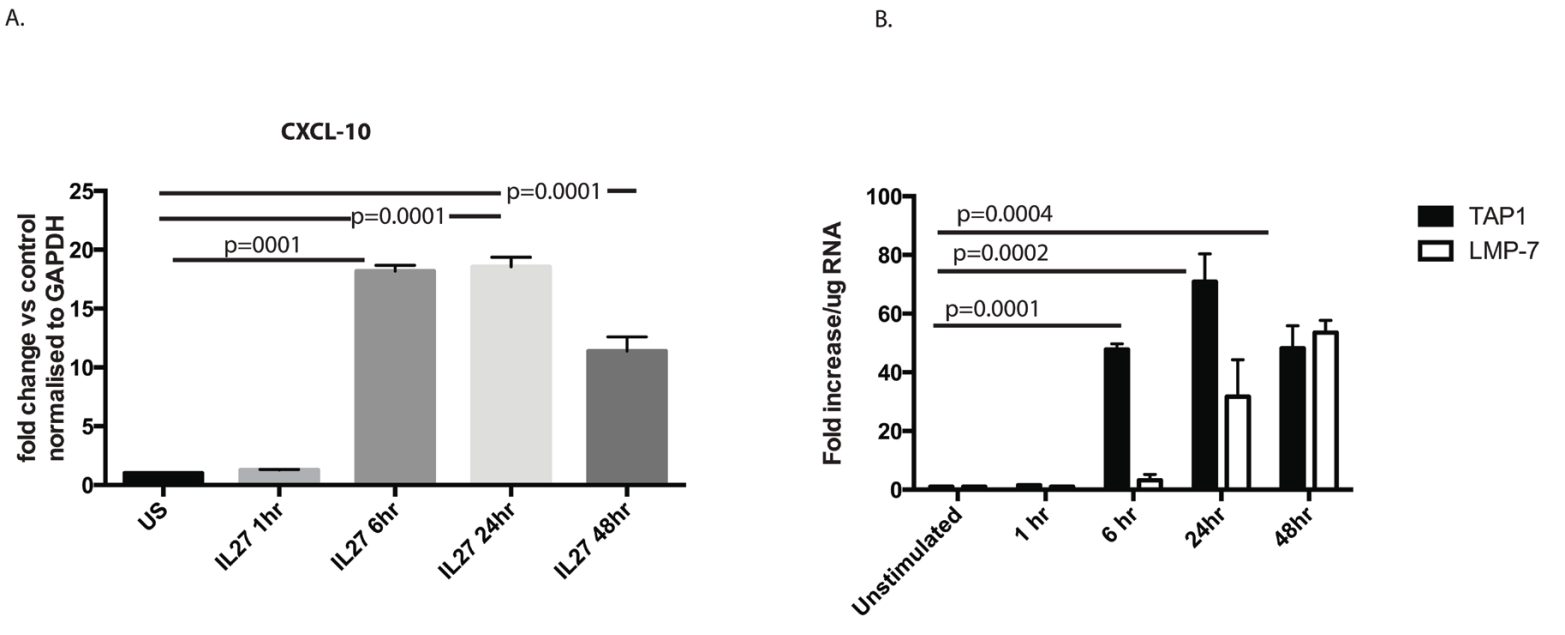
qPCR for CXCL-10, TAP1 and LMP7. ( **A**) IL-27 strongly induces CXCL-10 (IP-10) in hepatocytes. Huh7 cells were treated with IL-27 and quantitative PCR for CXCL-10 was performed over time (p value assessed using t test). ( **B**) mRNA expression of TAP1 and LMP7 in Huh7.5 cells treated with IL-27. Results are from three individual experiments. US, unstimulated; IL, interleukin; CXCL-10, chemokine 10; TAP1, transporter associated with antigen processing 1; LMP-7, low molecular mass polypeptide 7. Significant difference between values of unstimulated (US) and TAP1 were observed only after 6 hrs p=0.0001, and for US Vs LMP7 expression significant values were observed after 24 hrs of stimulation p=0.0132 and significance further increased at 48 hrs to p<0.0001.

One of the genes that was up-regulated by IL-27 was *CXCL-10* - an IFNγ-responsive gene. Therefore, as a test of principle and to reconfirm the results obtained by microarray analysis, we tested the level of gene expression of *CXCL10* in hepatocyte lines in response to IL-27. qPCR analysis showed that the levels of *CXCL-10* increased with time, reaching a peak at about 24 hrs post-stimulation (p=0.0001; Figure 3A).

Since proteasomal signaling emerged as an enriched pathway, *TAP1* and *LMP7*, important proteasomal genes involved in MHC class I presentation, were also examined by qPCR ^14^. We addressed this by analyzing the amount of *TAP1* in cells stimulated with IL-27 over time. qPCR analysis showed that the expression levels of *TAP1* increased from 6 hrs post stimulation (p=0.0001) and *LMP7* significantly increased increased from 24 hours (p=0.0132) post-stimulation with IL-27 ( Figure 3B).

## Discussion

IL-27 functions to activate diverse intracellular pathways in hepatocytes, but only a modest impact on viral replication was demonstrated in this study with HCVcc, and similarly for HBV. Consistent with this result, the gene expression dataset did not show a marked up-regulation of classical antiviral genes. This is different to previous findings using different cell models ^7^, where IL-27 has been shown to post-translationally modulate phosphorylation of *STAT1/3* in HepG2 cells. Other studies have also proposed that IL-27 might possess antiviral functions similar to IFNα ^5, 7, 15^. The fact that we did not observe a similar up-regulation of gene expression in our study could be due to differences in the cell lines used in the two studies. Consistent with this, in the above report another cell line within the same study did not show similar up-regulation of STAT phosphorylation ^7^. However, as suggested by others, IL-27 may synergize with other antiviral treatments, such as IFNα, for therapy for HBV ^16^. One further possible difference between studies was the time-point assessed – at 72hrs we may have missed some early antiviral gene expression, although overall the data is consistent with the lack of substantial impact of IL-27 in virus culture experiments over 1–2 weeks. We chose 72 hrs based on experience with other antiviral gene expression studies responses (e.g. IFN-lambda and alpha), and RNAseq studies in the HCVcc system, where we have used time-points up to day 10 ^17^.

Although we did not observe a clear antiviral gene set activated, we did observe some specific responses within the hepatocytes, such as up-regulation of *CXCL10, TAP1* and *LMP7*. These responses were confirmed as relevant by comparison with other datasets using GSEA, which revealed consistent patterns of response in diverse cell types, in response to related cytokines. *CXCL-10* is associated with the expression of IFNγ, but can be induced by other cytokines, and it has been shown that IL-27 is able to induce *CXCL-10* in bronchial epithelial cells ^18^. In skin tissue, antagonism of IL-27 attenuated the up-regulation of IFNγ, *CXCL-9, CXCL-10, CXCL-11* and tumor necrosis factor α mRNA ^19^. Our observation, stimulation with IL-27 up-regulated *CXCL-10*, is consistent with previous *in vitro* studies of hepatocytes ^7^.

The overall impact of IL-27 *in vivo* is still unclear. IL-27 has been observed to increase in patients with chronic HBV ^20^, and has also been shown to be able to modulate immune responses to prevent hepatic injury ^21^. Our *in vitro* data indicate it may have a potential impact on hepatocyte chemokine secretion and MHC class I antigen presentation, and thus *in vivo* studies of the role of IL-27 in modulating hepatocyte interaction with host CD8+ T cell responses may be of value in future.

## Data availability

IL-27 control dataset available from NCBI GEO (accession number, GSE89610;

https://www.ncbi.nlm.nih.gov/geo/query/acc.cgi?acc=GSE89610)

## Supplementary files

**Supplementary Table 1:** List of genes differentially regulated in response to 100ng/ml IL-27 at 72 hrs post stimulation.

**Supplementary Table 2:** (A) Kegg functional annotation of genes up-regulated in response to IL-27 in Huh7.5 cells by DAVID software analysis. (B) Gene functional annotation of genes up-regulated in response to IL-27 in Huh7.5 cells by DAVID software analysis.

**Supplementary Table 3:** (A) Comparative gene sets on immunological signatures from the MSigDB on genes up-regulated genes in response to IL-27. (B) Comparative Gene sets on immunological signatures from the MSigDB on genes down-regulated genes in response to IL-27.
