# Supplementary material for: Impact of IL-27 on hepatocyte antiviral gene expression and function
Source: Wellcome Open Res. 2016 Nov 17;1:17. [Version 1] doi: 10.12688/wellcomeopenres.9917.1 (PMC5207303; doi:10.12688/wellcomeopenres.9917.1)
Supplement: Peer review history

# Peer review: Referee response for version 1

**DOI:** 10.21956/wellcomeopenres.10688.r18168

**Published:** 2016-12-19

**Authors:** Ruth Broering

**Affiliations:** 1 Department of Gastroenterology and Hepatology, University Hospital of Essen, University of Duisburg-Essen, Essen, Germany

Ramamurthy et al. aimed to investigate the antiviral effect of IL-27 on hepatotrophic viruses (HCV, HBV) using HuH7.5 and HepaRG cell culture models. They observed a pro-inflammatory response but only modest antiviral effects. Although the findings are interesting there are several limitations regarding the experimental design.

- The choice of IL-27 dosing as well as the treatment duration is unclear; rationales need to be given here.
- The immunofluorescent staining in Figure 1 needs a DAPI signal to indicate HCV negative cells.
- There are discrepancies between the text and the Figure. Page 4: “… however, the effect is overall limited compared to the IFNγ positive control (Figure 1B) and was not significantly different when measured at a later time point (day 13).” There is no IFNγ mentioned in Figure 1.
- The HBV data given in Figure 1D are insufficient to draw any conclusions on IL-27 treatment. These data are of minor quality and need to be complemented (HBeAg, HBV DNA) or discarded.
- The number of replicates and repetitions within diverse experiments is not clear, especially for the gene array. Furthermore, the validation was performed with a single gene (CXCL-10) at a diverse time point.
- The links in the legend of Figure 2 seemed to be wrong. No IL-15 treatment is mentioned there.
- Drawing conclusions on antiviral responses of hepatocytes, while using Huh7 hepatoma cell line, is false. Here only suggestions can be made.

I have read this submission. I believe that I have an appropriate level of expertise to confirm that it is of an acceptable scientific standard, however I have significant reservations, as outlined above.

# Peer review: Referee response for version 1

**DOI:** 10.21956/wellcomeopenres.10688.r17986

**Published:** 2016-12-05

**Authors:** Andrew B. Lloyd

**Affiliations:** 1 Viral immunology Systems Program, Kirby Institute, University of New South Wales, Sydney, NSW, Australia

This in vitro study by Ramamurthy et al. evaluated the in vitro effects of IL-27 in Huh 7.5 HCVcc and HepaRG HBV cell culture models. Limited antiviral effect was demonstrated. The scope of the experiments was modest: a single concentration of IL-27 was utilised; (presumably) simultaneous inoculation of virus and administration of IL-27 only was utilised (as opposed to pre-treatment with IL-27); limited timepoints were analysed with 72 hours being the earliest. The read-outs in virus production were appropriate. The gene expression studies by microarray appear robust (including limited validation by qPCR for CXCL10) but provided unsurprising results.

I have read this submission. I believe that I have an appropriate level of expertise to confirm that it is of an acceptable scientific standard.

# Peer review: Referee response for version 1

**DOI:** 10.21956/wellcomeopenres.10688.r17744

**Published:** 2016-11-24

**Authors:** Shyamasundaran Kottilil

**Affiliations:** 1 Institute of Human Virology, University of Maryland, Baltimore, MD, USA

Ramamurthy et al. have evaluated the in vitro effects of IL-27, a novel cytokine, belonging to the IL-12 family using in vitro infectious HCV and HBV models. The results demonstrated a pro inflammatory rather than antiviral effect of IL-27.

  

1. The in vitro culture systems are excellent in evaluating direct antiviral effects of biologics and drugs. However, it does not allow us to quantitate the indirect effects (on T cells and other immune cells). Hence, based on these results, we cannot assume IL-27 has no antiviral effect mediated via immune cell potentiation, as it is expected to.
2. Please explain the rationale for selecting 100ng dose for IL-27. It would have been preferable to perform a dose ranging studies to better inform of antiviral effect.
3. Why was IFN-gamma used as positive control? Why not, IFN-alpha? There are more validated data about IFN-alpha mediated suppression of HCV in vitro than IFN-gamma.
4. IL-27 receptor expression on target cells was not evaluated in the study. This could be an important factor in determining what cell types respond to IL-27. Ideally, the measuring the density of the receptor on the cells would be beneficial to rule out cell line specific factors for discrepancy in the results.
5. The results shown here for HCV are quite different from previous published reports, some of which have shown significant effect on HCV replication. This study has evaluated HCV replication by PCR and IF and demonstrates no effect on HCV replication. The reasons for such discrepancy are not clear. Cell lines could have acquired or lost characteristics over time and could behave differently. Do we know the genotype of this cell line? Is it IL28B CC or TT?
6. Gene expression results shown in Figure 2 shows overlapping patterns with IL-15 induced gene expression in CD8 and NK cells. I am not sure what this means since gene expression in different cell types are likely to be controlled by cell specific transcriptional patterns and not comparable to effects of different cytokine on completely different cell types. This would benefit form addition of a description that will explain the results better.
7. Also, what was the rationale to choose 72 hours post treatment for gene expression analysis? Earlier studies have shown immediate induction of ISGs with Interferons and other antiviral cytokines.
8. Cell type specific expression of ISGs have been associated with favorable responses to interferon-alpha in patients. For example, higher expression of ISGs in hepatocyte is associated with a poor response to IFN-alpha, while a higher expression of ISGs in monocytes is associated with SVR. Hence, a lack of ISG expression in this hepatocyte cell line may be associated with an overall favorable host ISG response. Effects of IL-27 on other cell types may provide clues on the net effect of this cytokine on host innate response to HCV. 

I have read this submission. I believe that I have an appropriate level of expertise to confirm that it is of an acceptable scientific standard, however I have significant reservations, as outlined above.
